# Causal relationship between COVID-19 and myocarditis or pericarditis risk: a bidirectional Mendelian randomization study

**DOI:** 10.3389/fcvm.2023.1271959

**Published:** 2023-12-14

**Authors:** Guihong Liu, Tao Chen, Xin Zhang, Binbin Hu, Huashan Shi

**Affiliations:** ^1^ Department of Biotherapy, State Key Laboratory of Biotherapy, Cancer Center, West China Hospital, Sichuan University, Chengdu, China; ^2^ Department of Cardiology, The First Affiliated Hospital of China Medical University, Shenyang, China

**Keywords:** bidirectional two-sample Mendelian randomization, COVID-19, myocarditis, pericarditis, causal effect

## Abstract

**Background & aims:**

Coronavirus disease 2019 (COVID-19) is strongly associated with myocarditis or pericarditis risk in observational studies, however, there are still studies that do not support the above conclusion. Whether the observed association reflects causation needs to be confirmed. We performed a bidirectional Mendelian randomization (MR) study to assess the causal relationship of COVID-19, which was divided into three groups, namely severe COVID-19, hospitalized COVID-19, and COVID-19 infection, measured by myocarditis or pericarditis.

**Methods:**

We extracted summary genome-wide association statistics for the severe COVID-19 (case: 13,769, control: 1,072,442), hospitalized COVID-19 (case: 32,519, control: 2,062,805), COVID-19 infection (case: 122,616, control: 2,475,240), myocarditis (case 1,521, control 191,924), and pericarditis (case 979, control 286,109) among individuals of European ancestry. Independent genetic variants that exhibited a significant association with each phenotype at the genome-wide level of significance were utilized as instrumental variables. Estimation of the causal effect was mainly performed using the random effects inverse-variance weighted method (IVW). Additionally, other tests such as MR-Egger intercept, MR-PRESSO, Cochran's *Q*-test, “Leave-one-out”, and funnel plots were conducted to assess the extent of pleiotropy and heterogeneity.

**Results:**

Non-associations in the IVW and sensitivity analyses were observed for COVID-19 with myocarditis or pericarditis. Severe COVID-19 was not associated with myocarditis [odds ratio (OR), 1.00; 95% confidence interval (CI), 0.89–1.12; *P* = 0.99], pericarditis (OR = 0.90, 95% CI, 0.78–1.04, *P* = 0.17). Similar results can be observed in hospitalized COVID-19, and COVID-19 infection. At the same time, null associations were observed for myocarditis or pericarditis with COVID-19 traits in the reverse direction. The main results are kept stable in the sensitivity analysis.

**Conclusion:**

There is no evidence that COVID-19 is independently and causally associated with myocarditis or pericarditis.

## 1. Introduction

Coronavirus disease 2019 (COVID-19) is an epidemic disease caused by SARS-CoV-2, which is a sizable enveloped RNA virus, exhibiting approximately 80% sequence similarity with SARS-CoV, and predominantly impacts the respiratory system owing to its main attachment to angiotensin converting enzyme-2 (ACE-2) receptors (1, 2). SARS-CoV-2 can also commonly affect the cardiovascular, brain, kidney, liver, and immune systems, leading to their compromise (3). Myocarditis, pericarditis, acute coronary syndrome, arrhythmias, cardiomyopathy; heart failure, and thromboembolic disease are common cardiovascular damage manifestations of COVID-19 (2, 4).

Myocarditis is an inflammatory disease of the cardiac muscle, mainly diagnosed by histological, immunological, and immunohistochemical criteria (5). Pericarditis the most common form of pericardial disease refers to the inflammation of the pericardial layers (6). The etiology of myocarditis and pericarditis includes infectious and non-infectious forms. Viral infection and postviral immune-mediated responses are the most common causes of myocarditis (5). At the same time, viral infection is also a common cause of pericarditis. Sometimes myocarditis and pericarditis caused by viral infection can coexist (6, 7). According to some reports, myocarditis or pericarditis is a common cardiac complication caused by COVID-19 (8–10). During the COVID-19 pandemic, a study conducted by the Centers for Disease Control and Prevention (CDC) observed that the incidence of myocarditis infected with COVID-19 is 15 times that of non-infected COVID-19 (11). In European and American countries, it is estimated that the annual morbidity of acute pericarditis in the general population is about 27.7 cases per 100,000 people (12, 13). However, in the context of COVID-19 infection, real-world data demonstrate that new-onset pericarditis developed in approximately 1.5% of cases (14). More attention has been paid to myocarditis or pericarditis during the prevalence of COVID-19. Although there is growing evidence suggesting a potential association between COVID-19 and myocarditis and pericarditis, the precise causal relationship has not been definitively established. To date, limited reports with pathological evidence have demonstrated direct myocardial or pericardium invasion by the SARS-CoV-2 virus (15, 16). It is notable that even three years after the COVID-19 epidemic, our understanding of the pathogenesis of myocardial or pericardium injury in relation to COVID-19 remains incomplete. Several experts suggest that systemic inflammation may be the primary cause of myocardial injury, rather than direct viral infection of the heart (4, 17). The potential for long-term evolution into forms of inflammatory cardiomyopathy remains also unclear (18). Therefore, the relationship between COVID-19 and myocarditis and pericarditis still deserves further study, especially new research protocols, such as Mendelian randomization (MR).

Clinical research is susceptible to confounding factors and reverse causation. MR as a new and promising approach uses genetic variants as instrumental variables (IVs) to assess the genetic relationship between two phenotypes while avoiding potential confounding factors and reverse causation (19), which can be used to reveal the genetic sensitivity between COVID-19 and myocarditis or pericarditis. The reverse direction MR analysis was also performed (i.e., evaluating the causal association between myocarditis or pericarditis on COVID-19).

## 2. Materials and methods

### 2.1. Study design

**Figure 1:**
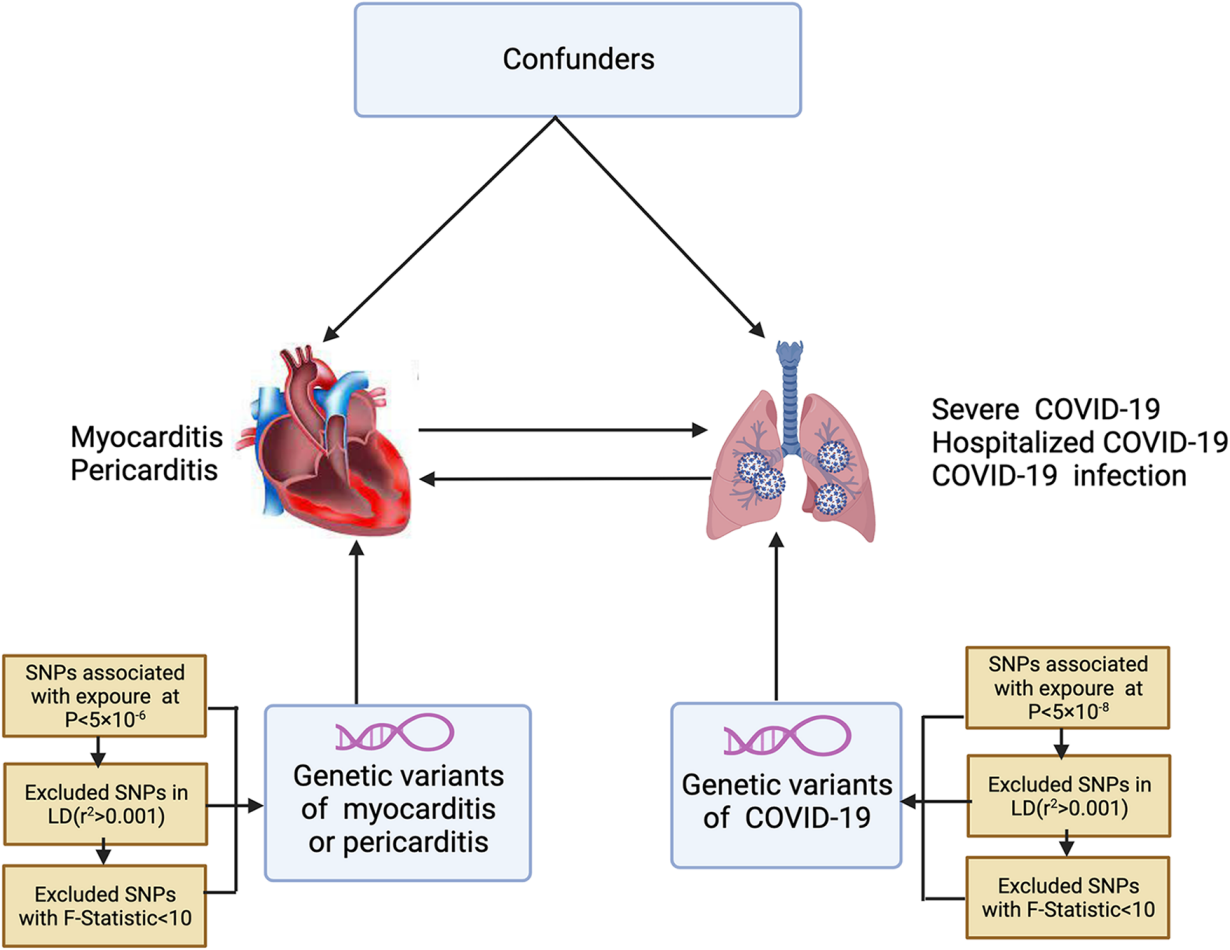
The conceptual framework for the bidirectional Mendelian randomization analysis. SNPs, single nucleotide polymorphisms; LD, linkage disequilibrium.

In Figure 1, the study design and the three indispensable assumptions of MR are portrayed, which are: (1) a robust association exists between single nucleotide polymorphisms (SNPs) and COVID-19; (2) SNPs are unaffected by any known confounding variables; and (3) the development of myocarditis or pericarditis is exclusively influenced by the association between SNPs and COVID-19. This assumption is also applicable in the reverse direction.

### 2.2. Data sources

In this two-sample MR study, the genetic association is the causal relationship between host genetic liability to COVID-19 on myocarditis or pericarditis. The exposures were severe COVID-19 (case: 13,769, control: 1,072,442), hospitalized COVID-19 (case: 32,519, control: 2,062,805), and COVID-19 infection (case: 122,616, control: 2,475,240). The outcomes were myocarditis (case 1,521, control 191,924) or pericarditis (case 979, control 286,109). The reverse MR analysis evaluates the possibility of reverse causal relationships. Myocarditis or pericarditis was treated as exposure, while COVID-19 events were treated as the outcome. Genome-wide association studies (GWAS) summary statistics of COVID-19 were obtained from the COVID-19 Host Genetic Initiative (HGI) (Round 7). Summary statistics of myocarditis or pericarditis were extracted from a GWAS conducted in finngen_R9_I9.

### 2.3. Selection and validation of SNPs

To identify suitable SNPs, we applied three criteria. First, we selected SNPs associated with COVID-19 at the genome-wide significance threshold with *P* < 5 × 10−8. In the reverse direction, independent instruments of myocarditis or pericarditis traits (*P* < 5 × 10−6) identified from the original GWAS were used as instruments. Second, we assessed the independence between selected SNPs using pairwise-linkage disequilibrium with a clumping window of 10,000 kb [22]. Third, the individual SNPs were assessed using the F-statistic to confirm their strength in mitigating potential biases. SNPs with F-statistics greater than ten were considered sufficiently powerful. Before conducting the MR analysis, data-harmonization measures were undertaken to ensure that the SNP's effects on exposure and outcome corresponded to the same allele.

### 2.4. MR analysis

An inverse-variance weighted (IVW) meta-analysis under a random-effect model was regarded as the primary analysis. MR-Egger, simple mode, weighted mode, and weighted median were used to complement IVW estimates. MR-Pleiotropy Residual Sum and Outlier methods (MR-PRESSO) were used to select abnormal SNPs. The intercept obtained from the MR-Egger regression was an index of directional pleiotropy. MR-PRESSO was also used to assess and correct horizontal pleiotropy (20). Cochrane's *Q*-value can detect heterogeneity. A leave-one-out sensitivity analysis was used to determine whether a single SNP affected the main causal relationship. Additionally, funnel plots assess whether causal associations are subject to potential bias. All statistical analyses used the “Two Sample MR” packages in R version 023.03.0 + 386.

## 3. Results

**Figure 2:**
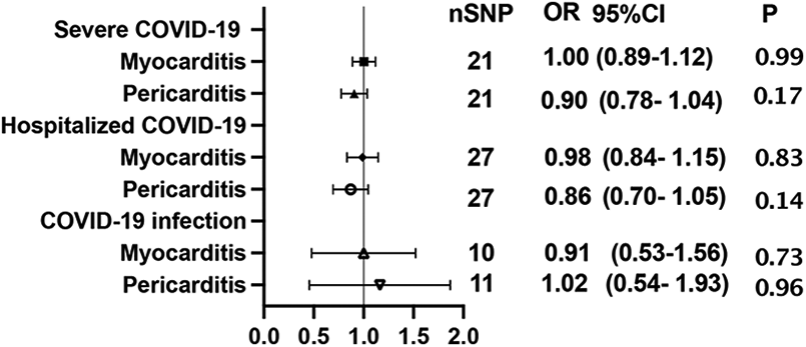
Mendelian randomization estimates the causal effect of COVID-19 on myocarditis or pericarditis. OR, odds ratio; CI, confidence interval.

**Figure 3:**
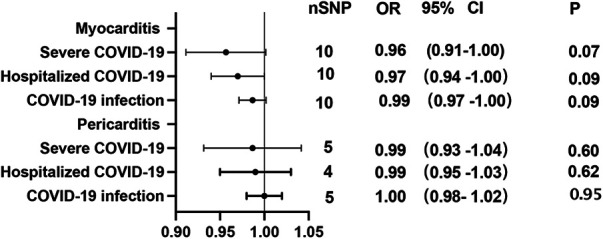
Mendelian randomization in the reverse direction estimates the causal effect of myocarditis or pericarditis on COVID-19. OR, odds ratio; CI, confidence interval.

Using GWAS summary statistics, the 2-sample MR analyses did not indicate any causal effect of genetically predicted COVID-19 on the risk of myocarditis or pericarditis in individuals of European descent. The *F*-values of individual SNP and total SNPs are greater than 10. IVW models indicated that genetically predicted severe COVID-19 was not associated with an increased risk of myocarditis (OR = 1.00, 95% CI, 0.89–1.12; *P* = 0.99), and pericarditis (OR = 0.90, 95% CI, 0.78–1.04, *P *= 0.17). Similar results can be observed in hospitalized COVID-19, and COVID-19 infection (Figure 2). At the same time, null associations were observed for myocarditis or pericarditis with COVID-19 traits (Figure 3). IVW models showed thatmyocarditis was not associated with an increased risk of severe COVID-19 (OR = 0.96, 95% CI, 0.91–1.00; *P* = 0.07), hospitalized COVID-19 (OR = 0.97, 95% CI, 0.94–1.00, *P* = 1.00), and COVID-infection (OR = 0.99, 95% CI, 0.97–1.00; *P* = 0.09). Similar results can be observed in pericarditis. No directional pleiotropic effect across the genetic variants was detected in the MR-Egger regression (Tables 1, 2). No horizontal pleiotropic effect across the genetic variants was detected in the MR-PRESSO (Tables 1, 2). The results of the IVW analysis did not suggest the existence of heterogeneity (Tables 1, 2). Associations of each variant with exposure and the risk of outcome are shown in Supplementary Tables S1–S12. The scatter plot for exposure on outcome was shown in Supplementary Figures S1–S12A. A forest plot of exposure on outcome was shown in Supplementary Figures S1–S12B. The leave-one-out sensitivity analysis showed that the association between exposure and outcome was not substantially driven by any individual SNP (Supplementary Figures S1–S12C). The funnel plot was symmetrical around the IVW estimates, revealing that the causal association was less susceptible to potential bias (Supplementary Figures S1–S12D).

**Table 1:** MR analysis of COVID-19 (exposure) with myocarditis or pericarditis trait outcomes.

Outcome	Method	OR	OR_lci95	OR_uci95	pval	MR-Egger intercept	MR-PRESSO global test	Cochran's Q heterogeneity test
						pval	pval	pval
Severe COVID-19 (exposure)								
Myocarditis	MR Egger	0.95	0.78	1.15	0.61	0.53	0.92	0.91
	Weighted median	0.96	0.82	1.12	0.57			
	Inverse variance weighted	1.00	0.89	1.12	0.99			
	Simple mode	0.90	0.67	1.20	0.46			
	Weighted mode	0.95	0.81	1.11	0.51			
Pericarditis	MR Egger	0.99	0.78	1.26	0.95	0.96	0.92	0.35
	Weighted median	0.97	0.80	1.18	0.78			
	Inverse variance weighted	0.90	0.78	1.04	0.17			
	Simple mode	1.04	0.72	1.50	0.83			
	Weighted mode	0.97	0.80	1.19	0.80			
COVID-19 hospital (exposure)								
Myocarditis	MR Egger	0.94	0.71	1.24	0.65	0.69	0.73	0.70
	Weighted median	0.93	0.74	1.17	0.55			
	Inverse variance weighted	0.98	0.84	1.15	0.83			
	Simple mode	0.88	0.57	1.38	0.59			
	Weighted mode	0.92	0.72	1.18	0.53			
Pericarditis	MR Egger	0.96	0.68	1.37	0.84	0.43	0.97	0.96
	Weighted median	0.95	0.71	1.28	0.75			
	Inverse variance weighted	0.86	0.70	1.05	0.14			
	Simple mode	0.82	0.48	1.40	0.47			
	Weighted mode	0.93	0.70	1.25	0.64			
COVID-19 infection (exposure)								
Myocarditis	MR Egger	0.54	0.21	1.40	0.24	0.23	0.57	0.63
	Weighted median	0.74	0.37	1.49	0.40			
	Inverse variance weighted	0.91	0.53	1.56	0.73			
	Simple mode	0.65	0.23	1.86	0.44			
	Weighted mode	0.65	0.28	1.52	0.35			
Pericarditis	MR Egger	0.94	0.29	3.00	0.92	0.87	0.89	0.89
	Weighted median	1.07	0.47	2.40	0.88			
	Inverse variance weighted	1.02	0.54	1.93	0.96			
	Simple mode	1.48	0.47	4.63	0.52			
	Weighted mode	0.98	0.38	2.54	0.97			

OR, odds ratio; LCI, low confidence interval; UCI, upper confidence interval.

**Table 2:** The reverse direction MR analysis evaluates the association of myocarditis or pericarditis with COVID-19 traits.

Outcome	Method	OR	OR_lci95	OR_uci95	pval	MR-Egger intercept	MR-PRESSO global test	Cochran's Q heterogeneity test
						pval	pval	pval
Myocarditis (exposure)								
Severe COVID-19	MR Egger	0.92	0.83	1.02	0.14	0.42	0.91	0.90
	Weighted median	0.96	0.90	1.02	0.17			
	Inverse variance weighted	0.96	0.91	1.00	0.07			
	Simple mode	0.95	0.87	1.04	0.32			
	Weighted mode	0.95	0.87	1.03	0.23			
Hospitalized COVID-19	MR Egger	0.95	0.89	1.01	0.15	0.42	0.64	0.62
	Weighted median	0.96	0.92	1.00	0.07			
	Inverse variance weighted	0.97	0.94	1.00	0.09			
	Simple mode	0.96	0.89	1.03	0.30			
	Weighted mode	0.96	0.90	1.02	0.25			
COVID-19 infection	MR Egger	1.00	0.98	1.03	0.80	0.17	0.32	0.32
	Weighted median	0.98	0.96	1.00	0.06			
	Inverse variance weighted	0.99	0.97	1.00	0.09			
	Simple mode	0.97	0.93	1.00	0.08			
	Weighted mode	0.97	0.94	1.00	0.08			
Pericarditis(expore)								
Severe COVID-19	MR Egger	1.02	0.93	1.11	0.72	0.43	0.75	0.72
	Weighted median	1.00	0.94	1.07	0.93			
	Inverse variance weighted	0.99	0.93	1.04	0.60			
	Simple mode	1.00	0.93	1.09	0.93			
	Weighted mode	1.00	0.93	1.09	0.91			
Hospitalized COVID-19	MR Egger	1.00	0.91	1.10	0.97	0.77	0.42	0.34
	Weighted median	1.00	0.94	1.05	0.89			
	Inverse variance weighted	0.99	0.95	1.03	0.62			
	Simple mode	1.00	0.94	1.07	0.99			
	Weighted mode	1.00	0.94	1.06	0.97			
COVID-19 infection	MR Egger	1.02	0.99	1.05	0.29	0.22	0.42	0.38
	Weighted median	1.00	0.98	1.02	0.81			
	Inverse variance weighted	1.00	0.98	1.02	0.95			
	Simple mode	1.00	0.97	1.04	0.94			
	Weighted mode	1.01	0.98	1.04	0.66			

OR, odds ratio; LCI, low confidence interval; UCI, upper confidence interval.

## 4. Discussion

This study conducted the first-ever 2-sample MR analysis examining the association between COVID-19 and myocarditis or pericarditis utilizing large-scale GWAS data. There is no evidence that COVID-19 is independently and causally associated with myocarditis or pericarditis.

Some studies indicated that during the COVID-19 pandemic, there is a substantial increase in the incidence of myocarditis and pericarditis. In a study from March 2020 to January 2021, the incidence of myocarditis was 0.146% among patients diagnosed with COVID-19 and 0.009% among patients without COVID-19, with a 15-fold difference compared to each other (11). In another study, among 718,365 patients diagnosed with COVID-19, 35,820 (5.0%) occurred myocarditis, and 10,706 (1.5%) occurred pericarditis (14). However, in a study, the incidence of myocarditis and pericarditis among 181,656 hospitalized COVID-19 patients was 0.08% (21). Another large study reported that among 277 postmortem examinations, the true incidence of myocarditis was likely less than 2% (22). A published meta-analysis about the rate of myocarditis in COVID-19 fatalities was also less than 2% (23). Our careful analysis of the above articles can conclude that the large variation in the incidence of COVID-19-associated myocarditis is due to diagnostic heterogeneity. The definitive diagnosis of myocarditis should be based on established histological and immunohistochemical (ICH) criteria with endomyocardial biopsy or autopsy (24). Due to the relatively lower rates of endomyocardial biopsies (EMBs) performed in patients with COVID-19, the diagnosis of myocarditis relies more extensively on evidence of myocardial injury or the use of cardiac magnetic resonance imaging (CMR) in conjunction with clinical symptoms (25, 26). These methods are employed to meet the criteria outlined in the CDC working definition of myocarditis. For example, in an Italy study, researchers conducted an autopsy series on 40 patients who had died from SARS-CoV-2 infection. The authors performed a thorough histological analysis of the heart tissues. Interestingly, only two out of the 40 patients exhibited indications of myocarditis, and only one patient had clear evidence of lymphocytic myocarditis. Notably, despite observing histological signs of lymphocytic infiltrate in three cases, the researchers did not detect the presence of the virus using reverse transcription polymerase chain reaction (RT-PCR), *in situ* hybridization (ISH), and IHC techniques (27). At the same time, the diagnosis of most patients with COVID-19 associated pericarditis is still based on symptoms and imaging examinations, but not pathological examinations (28, 29). Differences in diagnostic criteria have a greater impact on the incidence of myocarditis and pericarditis during the COVID-19 epidemic (20). We analyzed that this reason may significantly affect the result of this Mendelian analysis.

The mechanisms of COVID-19-associated myocarditis or pericarditis are complex and mainly include direct viral damage, cytokine storm, immune system dysregulation, and endothelial dysfunction (9, 30). Firstly, direct viral damage includes endocytosis of the virus and degradation of cellular structures, and overall myocyte injury (31, 32). Secondly, cytokine storm, as a well-known physiological mechanism associated with SARS-CoV-2 infection, leads to the excessive production of pro-inflammatory cytokines. This overproduction of pro-inflammatory cytokines has been linked to myocardial injury, as evidenced by elevated troponin levels observed during these conditions. Following the initial infection of the lungs by SARS-CoV-2, a cytokine storm is generated, characterized by the release of various cytokines such as tumor necrosis factor-alpha (TNFα), interleukin-1β (IL-1β), and IL-6. Alongside this, extracellular vesicles (EVs) carrying the virus or virus particles are also released. These EVs have the potential to travel through the bloodstream or lymphatic system to the heart, where they can infect cardiac cells expressing specific receptors such as ACE2, transmembrane serine protease-2 (TMPRSS2), and neuropilin-1 receptor (NRP1) (33). Thirdly, immune-mediated myocarditis is another important mechanism wherein both the innate and acquired immune responses lead to myocardial injury, ultimately leading to dilated cardiomyopathy. Autoimmune-mediated myocarditis can occur when cryptic antigens in cardiomyocytes, are released following virus-mediated injury. In cases where viral antigens successfully evade the innate immune system, they replicate and produce virus-associated proteins that directly cause myocardial injury by inducing apoptosis and necrosis. Similar to other viral pathogens, SARS-CoV-2 may induce myocarditis in humans through a similar pathway. Noteworthily, cytokine storm promotes the activation of T-cells and releases cytokines to maintain the exaggerated immune response (34). The fourth mechanism involves the direct entry of the virus into endothelial cells within the heart, without necessarily infecting myocytes. This direct infection of endothelial cells has been observed in autopsy hearts and glomerular endothelial cells, through the use of electron microscopy. However, in some cases, the appearance and location of these virus particles within cells were not typical of those observed in coronavirus-infected cells (30, 35).

Although myocarditis or pericarditis caused by COVID-19 may be related to the above mechanisms, there is still some controversy. First, some studies believed that the evidence for direct damage to myocardial cells caused by viruses is not sufficient. In a study involving 39 consecutive autopsy cases from Germany, the viral load of SARS-CoV-2 was quantified. The results showed that SARS-CoV-2 was detected in the hearts of 24 out of 39 cases (61.5%), with 16 out of 39 cases (41%) exhibiting viral copy numbers higher than 1,000 copies per microgram of RNA. Virus replication was observed in five patients with the highest viral load. However, ISH confirmed the presence of the virus in interstitial cells within the cardiac tissue, rather than in the myocytes themselves. Notably, the presence of the virus was not associated with increased infiltration of mononuclear cells into the myocardium, and no cases of myocarditis were identified according to the Dallas criteria (15). At the same time, other researchers also have not found a SARS-CoV-2 genome detected within the myocardium (16, 36). Second, drug-induced myocarditis should also be considered because it has been confirmed in autopsy reports of COVID-19 deaths receiving antivirals (37, 38). Third, Takotsubo cardiomyopathy and myocarditis have similar CMR manifestations, mainly including injury to myocytes, and infiltration of lymphocytes and macrophages in the autopsied specimens. Some so-called COVID-19-related myocarditis cannot be ruled out as Takotsubo cardiomyopathy, especially in patients with a lack of evident myocardial SARS-CoV-2 viral genome (39).

Another concerning issue is confounding factors, such as immunodeficiency. A study showed an increased risk of more severe presentations and higher mortality caused by COVID-19 for patients with solid-organ transplants or cancer (40). According to a recent report, patients with cancer treated with checkpoint inhibitors were at particularly high risk of severe COVID-19 (41). Another study including 60 patients with primary immunodeficiency (PID) and 40 patients with secondary immunodeficiency (SID) included that, compared with the general population, adult patients with PID and symptomatic SID display greater morbidity and mortality from COVID-19 (42). A study concluded that myocarditis was found in 37 cases (52%) according to a retrospective evaluation of 71 consecutive necropsy patients who died from acquired immunodeficiency syndrome (AIDS) (43). Another study included that among 181 consecutive patients at all stages of HIV infection, 109 patients (60%) occurred different degrees of pericarditis (44). We could find that immunodeficiency as a confounding factor is not only associated with COVID-19 infection but also increases the incidence of myocarditis and pericarditis. Therefore, we cannot ignore that confounding factors have a great effect on the results of MR analysis.

Although there is already a large number of literature on the relationship between COVID-19 and myocarditis or pericarditis, our article still has outstanding novelty. Firstly, this is the first time that we have used a new research method—MR to clarify the causal relationship between them. Compared to traditional clinical studies, MR is less prone to potential confounding and avoids reverse causation because genetic variation is allocated at conception, and thus, it can strengthen the evidence for causal inference (45, 46). At the same time, in order to verify whether there is a reverse causal relationship, our article uses bidirectional MR. Secondly, our data comes from the latest GWAS database. The huge sample size and the latest data further improve the stability and credibility of the results. Finally, we conducted an assessment of pleiotropy, and heterogeneity and invited a professional from the data center to assist and verify our statistical and computational processes to ensure the stability and reliability of the results.

## 5. Limitation

Our study has several limitations. Firstly, most of the included patients with myocarditis or pericarditis were diagnosed by clinical symptoms and signs, auxiliary examinations (electrocardiogram, myocardial enzymes, cardiac color Doppler ultrasound, CMR, etc.), and no EMB or autopsy was performed. This may have a significant impact on our analysis. Secondly, myocarditis or pericarditis is equivalent to the number of COVID-19 patients being too small, and the screened SNPs are relatively insufficient. Finally, the majority of participants were of European ancestry. Hence, the generalizability of the results cannot be derived for other races.

## 6. Conclusion

Our 2-sample MR analysis did not yield any supportive genetic evidence for a causal association between COVID-19 and an elevated risk of myocarditis or pericarditis. Although growing evidence indicates a causal relationship between COVID-19 and myocarditis or pericarditis, we need to consider the diagnostic heterogeneity of myocarditis or pericarditis and the effect of confounders. According to our conclusions and other evidence, the relationship between COVID-19 and myocarditis or pericarditis still deserves further research and observation.

## Data Availability

The original contributions presented in the study are included in the article/**Supplementary Material**, further inquiries can be directed to the corresponding author.

## Supplementary material

Supplementary Figure S1, Supplementary Figure S2, Supplementary Figure S3, Supplementary Figure S4, Supplementary Figure S5, Supplementary Figure S6, Supplementary Figure S7, Supplementary Figure S8, Supplementary Figure S9, Supplementary Figure S10, Supplementary Figure S11, Supplementary Figure S12 (**A**) Regression lines of MR tests, (**B**) forrest plot of single SNP from hospitalized COVID-19 on myocarditis, (**C**) MR leave-one-out sensitivity analysis, (**D**) funnel of single SNP from hospitalized COVID-19 on myocarditis. (**A**) Regression lines of MR tests, (**B**) forrest plot of single SNP from hospitalized COVID-19 on myocarditis, (**C**) MR leave-one-out sensitivity analysis, (**D**) funnel of single SNP from hospitalized COVID-19 on pericarditis. (**A**) Regression lines of MR tests, (**B**) forrest plot of single SNP from hospitalized COVID-19 on myocarditis, (**C**) MR leave-one-out sensitivity analysis, (**D**) Funnel of single SNP from COVID-19 infection on myocarditis. (**A**) Regression lines of MR tests, (**B**) forrest plot of single SNP from hospitalized COVID-19 on myocarditis, (**C**) MR leave-one-out sensitivity analysis, (**D**) funnel of single SNP from COVID-19 infection on pericarditis. (**A**) Regression lines of MR tests, (**B**) forrest plot of single SNP from hospitalized COVID-19 on myocarditis, (**C**) MR leave-one-out sensitivity analysis, (**D**) funnel of single SNP from severe COVID-19 on myocarditis. (**A**) Regression lines of MR tests, (**B**) forrest plot of single SNP from hospitalized COVID-19 on myocarditis, (**C**) MR leave-one-out sensitivity analysis, (**D**) funnel of single SNP from severe COVID-19 on pericarditis. (**A**) Regression lines of MR tests, (**B**) forrest plot of single SNP from hospitalized COVID-19 on myocarditis, (**C**) MR leave-one-out sensitivity analysis, (**D**) Funnel of single SNP from myocarditis on hospitalized COVID-19. (**A**) Regression lines of MR tests, (**B**) forrest plot of single SNP from hospitalized COVID-19 on myocarditis, (**C**) MR leave-one-out sensitivity analysis, (**D**) funnel of single SNP from myocarditis on COVID-19 infection. (**A**) Regression lines of MR tests, (**B**) forrest plot of single SNP from hospitalized COVID-19 on myocarditis, (**C**) MR leave-one-out sensitivity analysis, (**D**) funnel of single SNP from myocarditis on severe COVID-19. (**A**) Regression lines of MR tests, (**B**) forrest plot of single SNP from hospitalized COVID-19 on myocarditis, (**C**) MR leave-one-out sensitivity analysis, (**D**) funnel of single SNP from pericarditis on hospitalized COVID-19. (**A**) Regression lines of MR tests, (**B**) forrest plot of single SNP from hospitalized COVID-19 on myocarditis, (**C**) MR leave-one-out sensitivity analysis, (**D**) funnel of single SNP from pericarditis on COVID-19 infection. (**A**) Regression lines of MR tests, (**B**) forrest plot of single SNP from hospitalized COVID-19 on myocarditis, (**C**) MR leave-one-out sensitivity analysis, (**D**) funnel of single SNP from pericarditis on severe COVID-19.

Supplementary Table S1, Supplementary Table S2, Supplementary Table S3, Supplementary Table S4, Supplementary Table S5, Supplementary Table S6, Supplementary Table S7, Supplementary Table S8, Supplementary Table S9, Supplementary Table S10, Supplementary Table S11, Supplementary Table S12 Association of the genetic instruments with severe COVID-19 and myocarditis. Association of the genetic instruments with severe COVID-19 and pericarditis. Association of the genetic instruments with hospitalized COVID-19 and myocarditis. Association of the genetic instruments with hospitalized COVID-19 and pericarditis. Association of the genetic instruments with COVID-19 infection and myocarditis. Association of the genetic instruments with COVID-19 infection and pericarditis. Association of the genetic instruments with myocarditis and hospitalized COVID-19. Association of the genetic instruments with myocarditis and COVID-19 infection. Association of the genetic instruments with myocarditis and severe COVID-19. Association of the genetic instruments with pericarditis and hospitalized COVID-19. Association of the genetic instruments with pericarditis and COVID-19 infection. Association of the genetic instruments with pericarditis and severe COVID-19.
