# Supplementary figures and images for: Causal relationship between COVID-19 and myocarditis or pericarditis risk: a bidirectional Mendelian randomization study
Source: Front Cardiovasc Med. 2023 Dec 14;10:1271959. doi: 10.3389/fcvm.2023.1271959 (PMC10755931; doi:10.3389/fcvm.2023.1271959)

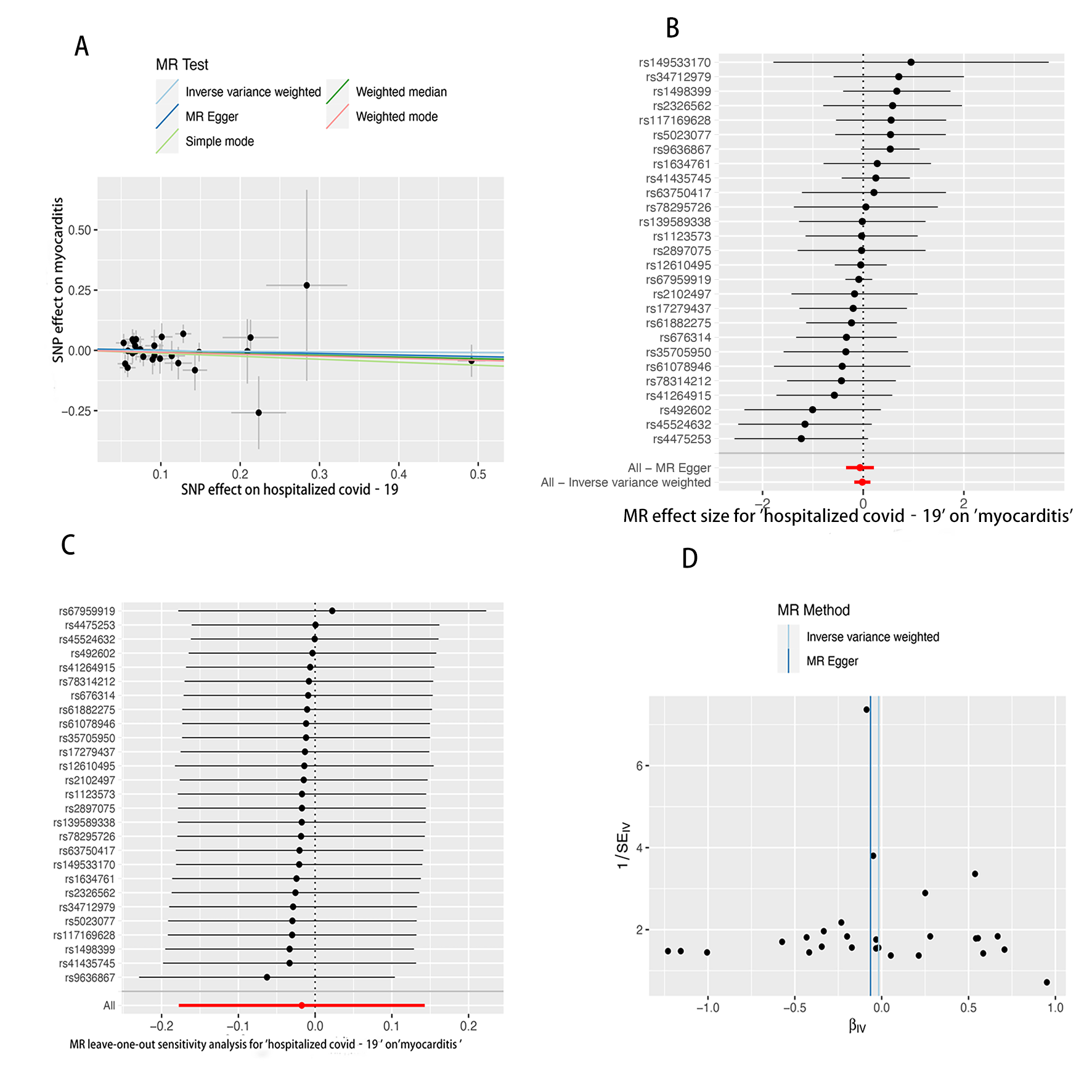

Supplement: Supplementary Table S1, Supplementary Table S2, Supplementary Table S3, Supplementary Table S4, Supplementary Table S5, Supplementary Table S6, Supplementary Table S7, Supplementary Table S8, Supplementary Table S9, Supplementary Table S10, Supplementary Table S11, Supplementary Table S12 — Association of the genetic instruments with severe COVID-19 and myocarditis. Association of the genetic instruments with severe COVID-19 and pericarditis. Association of the genetic instruments with hospitalized COVID-19 and myocarditis. Association of the genetic instruments with hospitalized COVID-19 and pericarditis. Association of the genetic instruments with COVID-19 infection and myocarditis. Association of the genetic instruments with COVID-19 infection and pericarditis. Association of the genetic instruments with myocarditis and hospitalized COVID-19. Association of the genetic instruments with myocarditis and COVID-19 infection. Association of the genetic instruments with myocarditis and severe COVID-19. Association of the genetic instruments with pericarditis and hospitalized COVID-19. Association of the genetic instruments with pericarditis and COVID-19 infection. Association of the genetic instruments with pericarditis and severe COVID-19. [file Datasheet2.zip › Data Sheet 2_v2/Data Sheet 2 (1)/Supplementary figures (1-12)/Figure 1.tif]

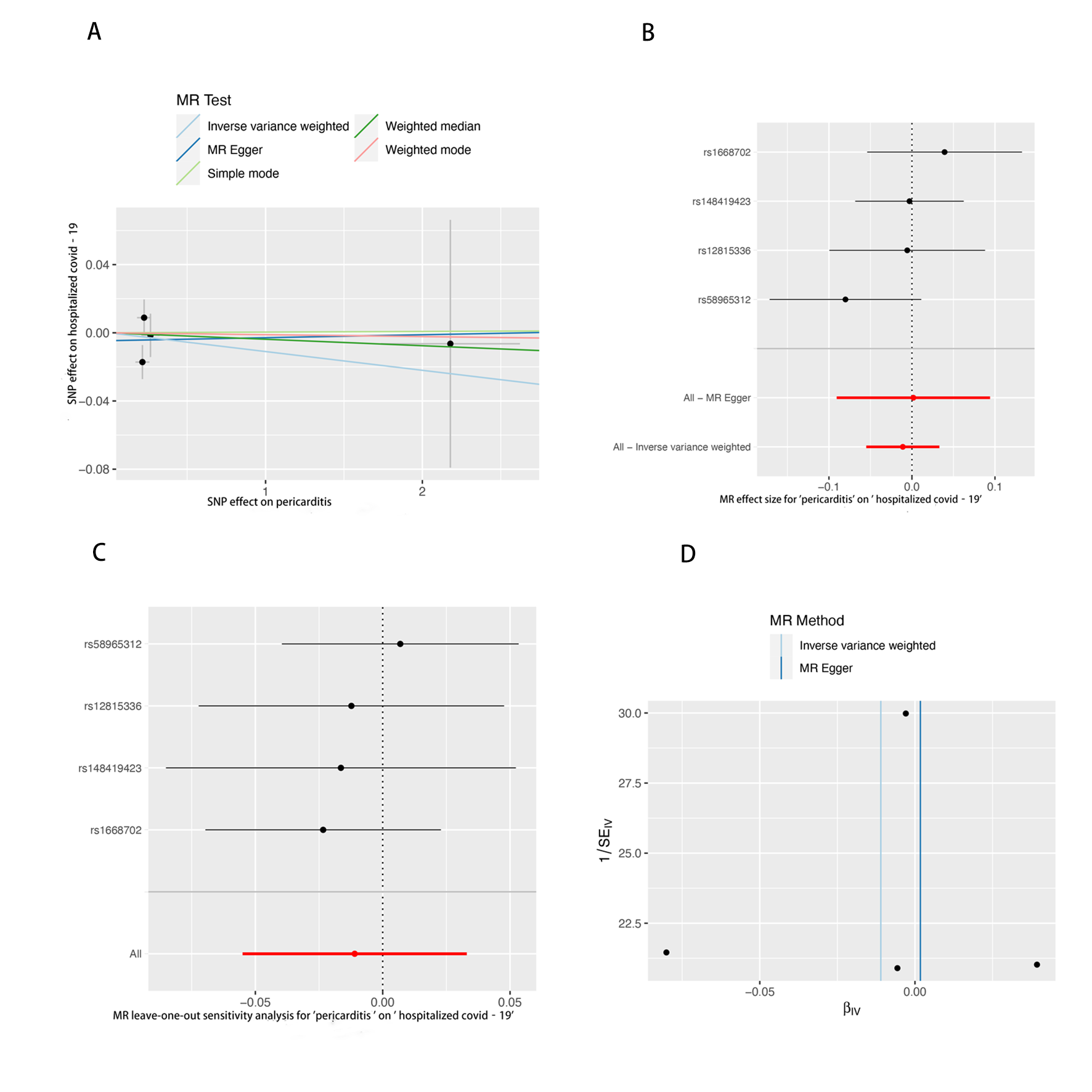

Supplement: Supplementary Table S1, Supplementary Table S2, Supplementary Table S3, Supplementary Table S4, Supplementary Table S5, Supplementary Table S6, Supplementary Table S7, Supplementary Table S8, Supplementary Table S9, Supplementary Table S10, Supplementary Table S11, Supplementary Table S12 — Association of the genetic instruments with severe COVID-19 and myocarditis. Association of the genetic instruments with severe COVID-19 and pericarditis. Association of the genetic instruments with hospitalized COVID-19 and myocarditis. Association of the genetic instruments with hospitalized COVID-19 and pericarditis. Association of the genetic instruments with COVID-19 infection and myocarditis. Association of the genetic instruments with COVID-19 infection and pericarditis. Association of the genetic instruments with myocarditis and hospitalized COVID-19. Association of the genetic instruments with myocarditis and COVID-19 infection. Association of the genetic instruments with myocarditis and severe COVID-19. Association of the genetic instruments with pericarditis and hospitalized COVID-19. Association of the genetic instruments with pericarditis and COVID-19 infection. Association of the genetic instruments with pericarditis and severe COVID-19. [file Datasheet2.zip › Data Sheet 2_v2/Data Sheet 2 (1)/Supplementary figures (1-12)/FIgure 10.tif]

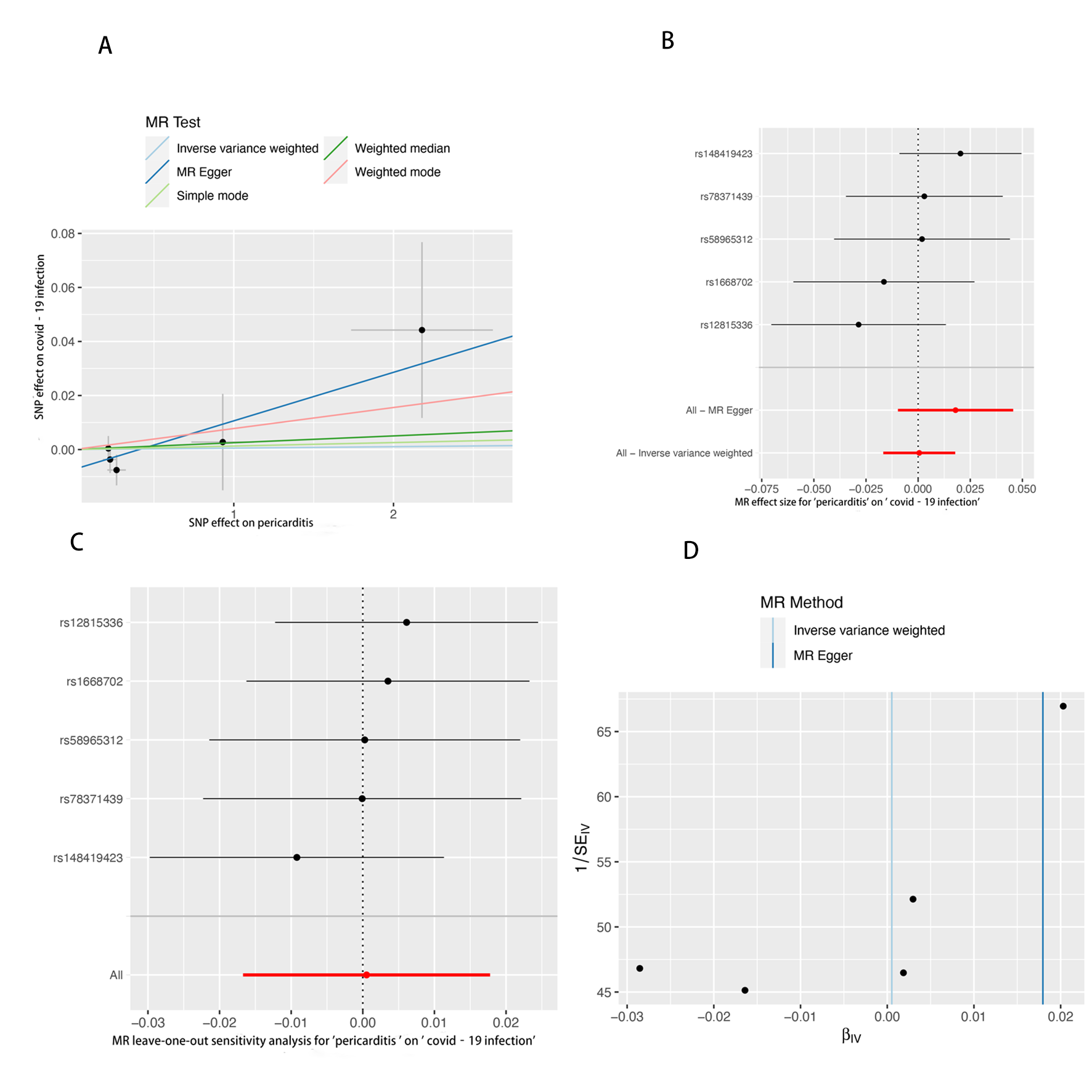

Supplement: Supplementary Table S1, Supplementary Table S2, Supplementary Table S3, Supplementary Table S4, Supplementary Table S5, Supplementary Table S6, Supplementary Table S7, Supplementary Table S8, Supplementary Table S9, Supplementary Table S10, Supplementary Table S11, Supplementary Table S12 — Association of the genetic instruments with severe COVID-19 and myocarditis. Association of the genetic instruments with severe COVID-19 and pericarditis. Association of the genetic instruments with hospitalized COVID-19 and myocarditis. Association of the genetic instruments with hospitalized COVID-19 and pericarditis. Association of the genetic instruments with COVID-19 infection and myocarditis. Association of the genetic instruments with COVID-19 infection and pericarditis. Association of the genetic instruments with myocarditis and hospitalized COVID-19. Association of the genetic instruments with myocarditis and COVID-19 infection. Association of the genetic instruments with myocarditis and severe COVID-19. Association of the genetic instruments with pericarditis and hospitalized COVID-19. Association of the genetic instruments with pericarditis and COVID-19 infection. Association of the genetic instruments with pericarditis and severe COVID-19. [file Datasheet2.zip › Data Sheet 2_v2/Data Sheet 2 (1)/Supplementary figures (1-12)/Figure 11.tif]

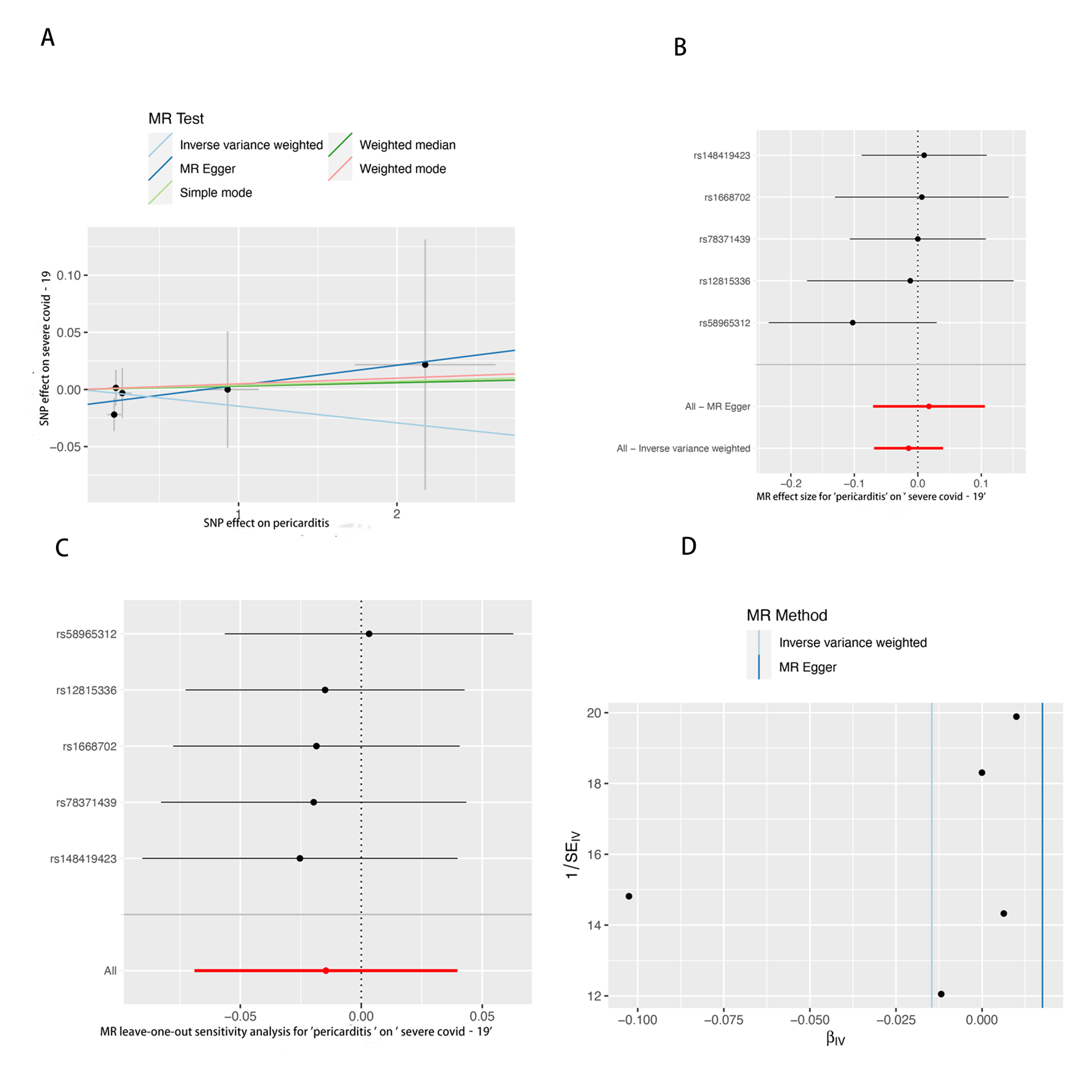

Supplement: Supplementary Table S1, Supplementary Table S2, Supplementary Table S3, Supplementary Table S4, Supplementary Table S5, Supplementary Table S6, Supplementary Table S7, Supplementary Table S8, Supplementary Table S9, Supplementary Table S10, Supplementary Table S11, Supplementary Table S12 — Association of the genetic instruments with severe COVID-19 and myocarditis. Association of the genetic instruments with severe COVID-19 and pericarditis. Association of the genetic instruments with hospitalized COVID-19 and myocarditis. Association of the genetic instruments with hospitalized COVID-19 and pericarditis. Association of the genetic instruments with COVID-19 infection and myocarditis. Association of the genetic instruments with COVID-19 infection and pericarditis. Association of the genetic instruments with myocarditis and hospitalized COVID-19. Association of the genetic instruments with myocarditis and COVID-19 infection. Association of the genetic instruments with myocarditis and severe COVID-19. Association of the genetic instruments with pericarditis and hospitalized COVID-19. Association of the genetic instruments with pericarditis and COVID-19 infection. Association of the genetic instruments with pericarditis and severe COVID-19. [file Datasheet2.zip › Data Sheet 2_v2/Data Sheet 2 (1)/Supplementary figures (1-12)/Figure 12.tif]

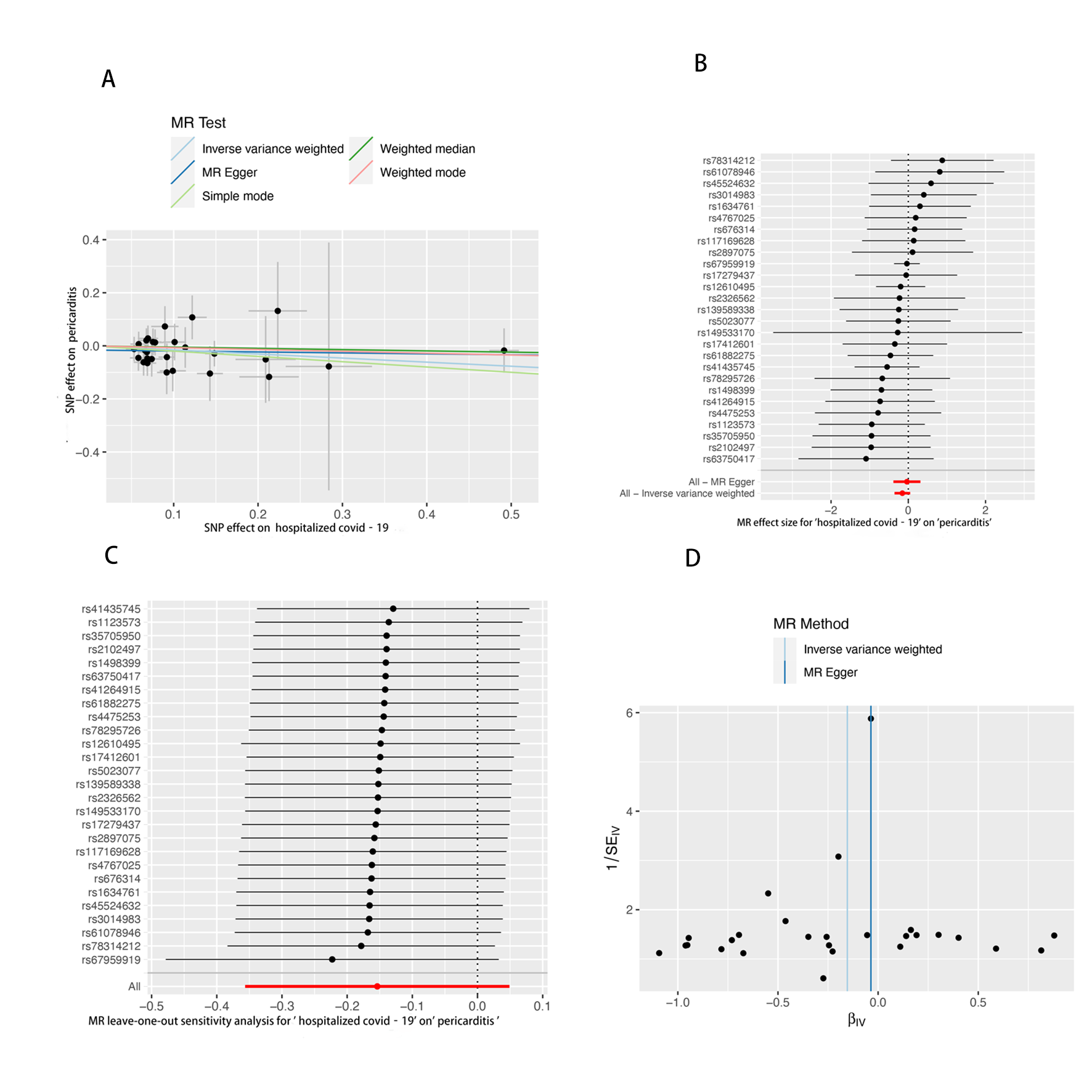

Supplement: Supplementary Table S1, Supplementary Table S2, Supplementary Table S3, Supplementary Table S4, Supplementary Table S5, Supplementary Table S6, Supplementary Table S7, Supplementary Table S8, Supplementary Table S9, Supplementary Table S10, Supplementary Table S11, Supplementary Table S12 — Association of the genetic instruments with severe COVID-19 and myocarditis. Association of the genetic instruments with severe COVID-19 and pericarditis. Association of the genetic instruments with hospitalized COVID-19 and myocarditis. Association of the genetic instruments with hospitalized COVID-19 and pericarditis. Association of the genetic instruments with COVID-19 infection and myocarditis. Association of the genetic instruments with COVID-19 infection and pericarditis. Association of the genetic instruments with myocarditis and hospitalized COVID-19. Association of the genetic instruments with myocarditis and COVID-19 infection. Association of the genetic instruments with myocarditis and severe COVID-19. Association of the genetic instruments with pericarditis and hospitalized COVID-19. Association of the genetic instruments with pericarditis and COVID-19 infection. Association of the genetic instruments with pericarditis and severe COVID-19. [file Datasheet2.zip › Data Sheet 2_v2/Data Sheet 2 (1)/Supplementary figures (1-12)/Figure 2.tif]

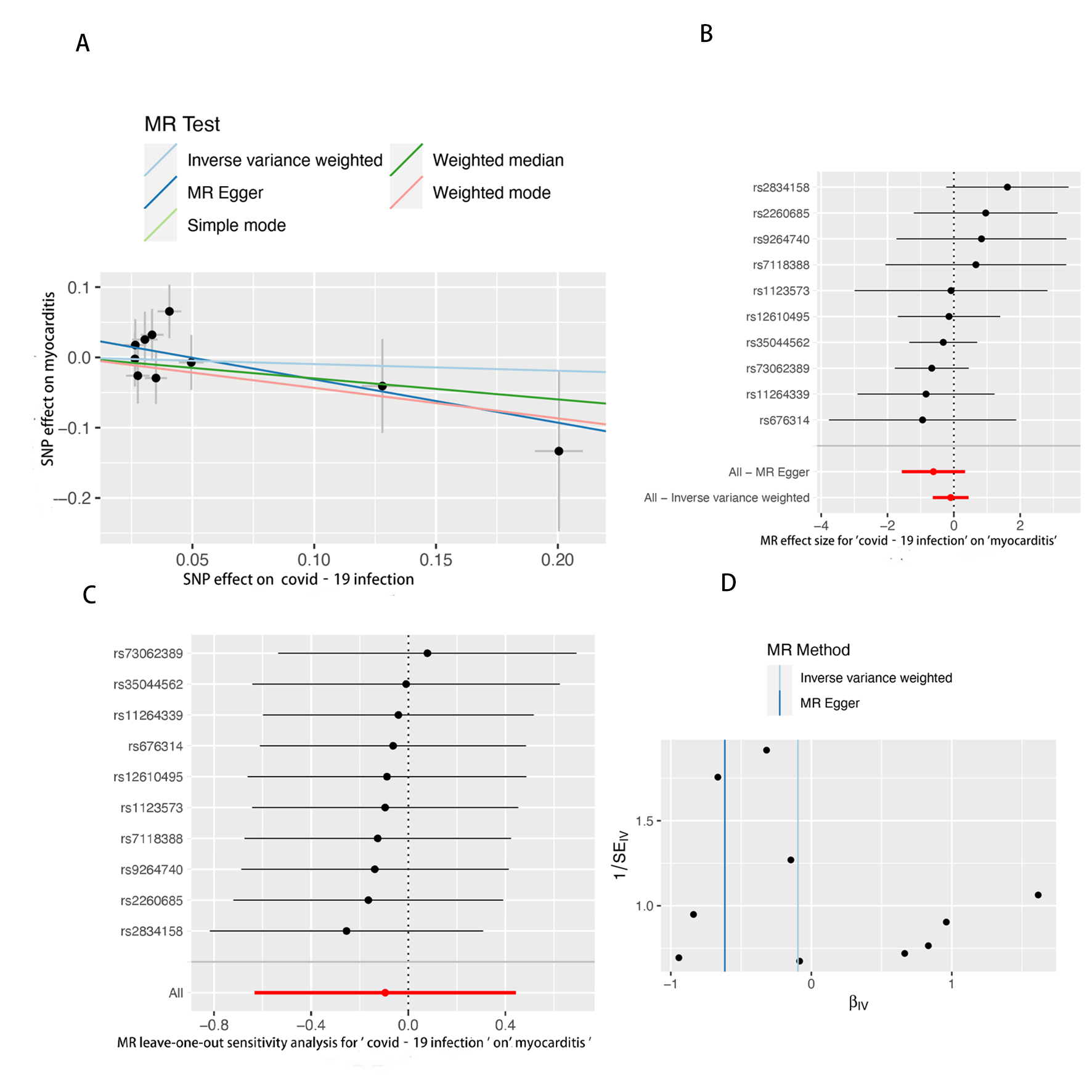

Supplement: Supplementary Table S1, Supplementary Table S2, Supplementary Table S3, Supplementary Table S4, Supplementary Table S5, Supplementary Table S6, Supplementary Table S7, Supplementary Table S8, Supplementary Table S9, Supplementary Table S10, Supplementary Table S11, Supplementary Table S12 — Association of the genetic instruments with severe COVID-19 and myocarditis. Association of the genetic instruments with severe COVID-19 and pericarditis. Association of the genetic instruments with hospitalized COVID-19 and myocarditis. Association of the genetic instruments with hospitalized COVID-19 and pericarditis. Association of the genetic instruments with COVID-19 infection and myocarditis. Association of the genetic instruments with COVID-19 infection and pericarditis. Association of the genetic instruments with myocarditis and hospitalized COVID-19. Association of the genetic instruments with myocarditis and COVID-19 infection. Association of the genetic instruments with myocarditis and severe COVID-19. Association of the genetic instruments with pericarditis and hospitalized COVID-19. Association of the genetic instruments with pericarditis and COVID-19 infection. Association of the genetic instruments with pericarditis and severe COVID-19. [file Datasheet2.zip › Data Sheet 2_v2/Data Sheet 2 (1)/Supplementary figures (1-12)/Figure 3.tif]

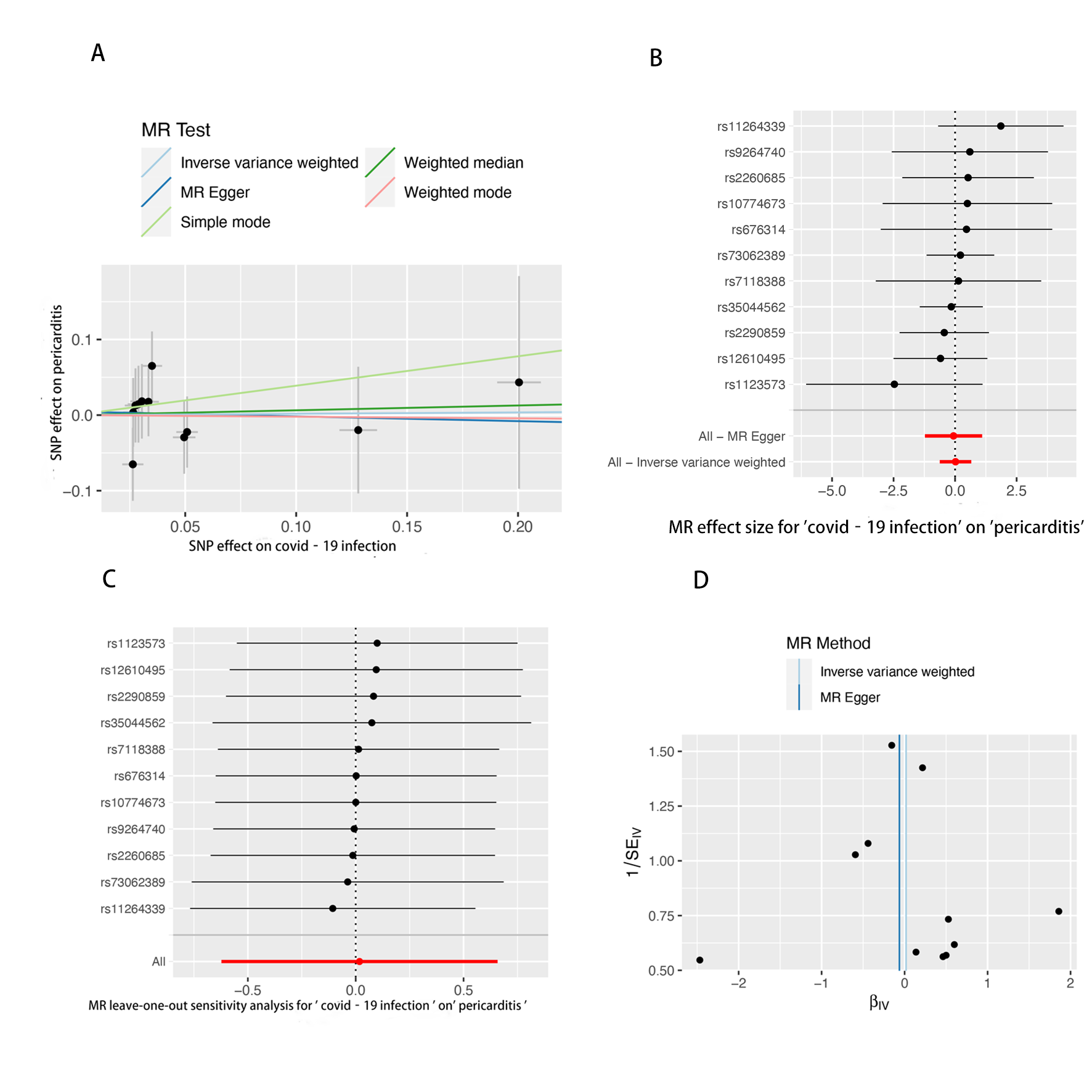

Supplement: Supplementary Table S1, Supplementary Table S2, Supplementary Table S3, Supplementary Table S4, Supplementary Table S5, Supplementary Table S6, Supplementary Table S7, Supplementary Table S8, Supplementary Table S9, Supplementary Table S10, Supplementary Table S11, Supplementary Table S12 — Association of the genetic instruments with severe COVID-19 and myocarditis. Association of the genetic instruments with severe COVID-19 and pericarditis. Association of the genetic instruments with hospitalized COVID-19 and myocarditis. Association of the genetic instruments with hospitalized COVID-19 and pericarditis. Association of the genetic instruments with COVID-19 infection and myocarditis. Association of the genetic instruments with COVID-19 infection and pericarditis. Association of the genetic instruments with myocarditis and hospitalized COVID-19. Association of the genetic instruments with myocarditis and COVID-19 infection. Association of the genetic instruments with myocarditis and severe COVID-19. Association of the genetic instruments with pericarditis and hospitalized COVID-19. Association of the genetic instruments with pericarditis and COVID-19 infection. Association of the genetic instruments with pericarditis and severe COVID-19. [file Datasheet2.zip › Data Sheet 2_v2/Data Sheet 2 (1)/Supplementary figures (1-12)/Figure 4.tif]

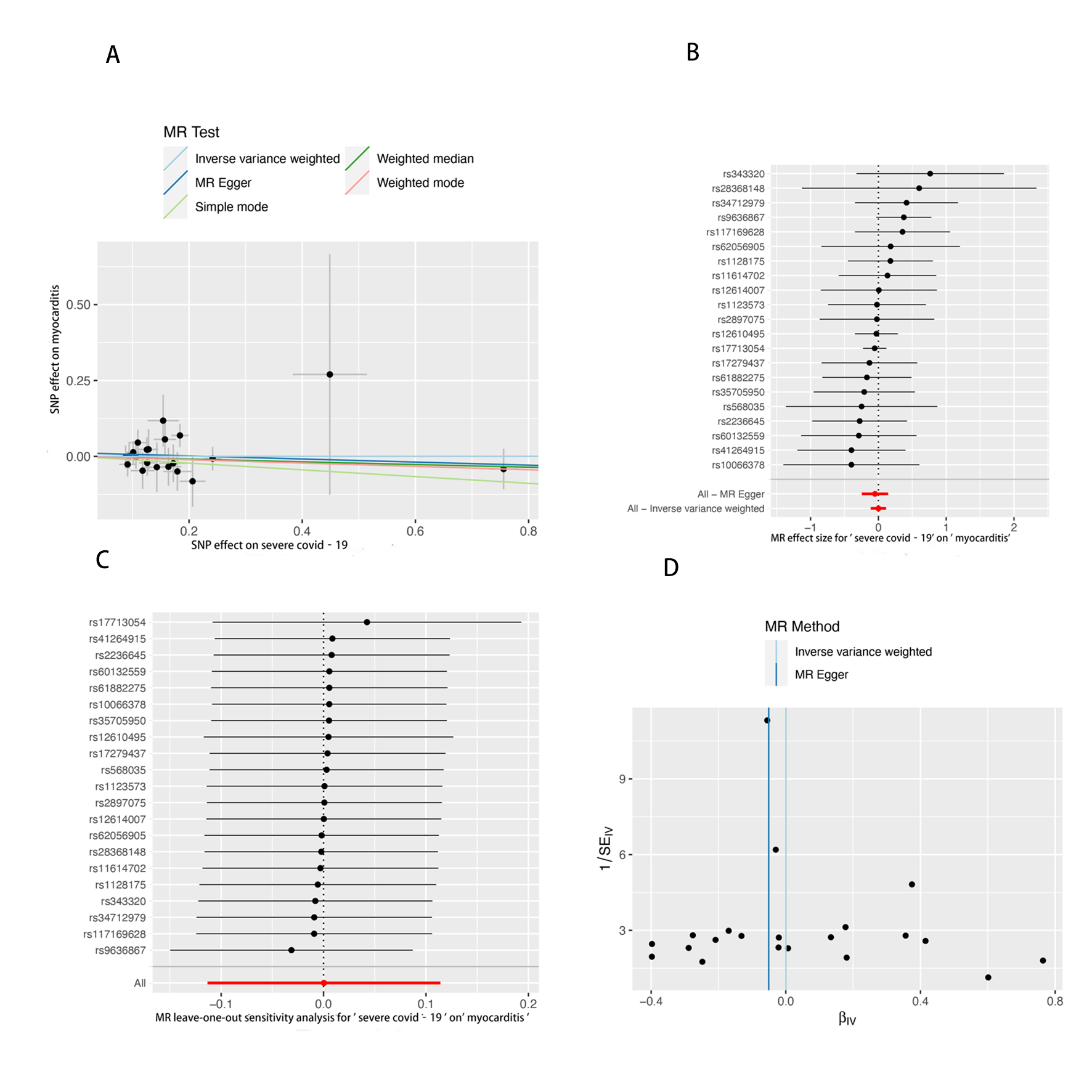

Supplement: Supplementary Table S1, Supplementary Table S2, Supplementary Table S3, Supplementary Table S4, Supplementary Table S5, Supplementary Table S6, Supplementary Table S7, Supplementary Table S8, Supplementary Table S9, Supplementary Table S10, Supplementary Table S11, Supplementary Table S12 — Association of the genetic instruments with severe COVID-19 and myocarditis. Association of the genetic instruments with severe COVID-19 and pericarditis. Association of the genetic instruments with hospitalized COVID-19 and myocarditis. Association of the genetic instruments with hospitalized COVID-19 and pericarditis. Association of the genetic instruments with COVID-19 infection and myocarditis. Association of the genetic instruments with COVID-19 infection and pericarditis. Association of the genetic instruments with myocarditis and hospitalized COVID-19. Association of the genetic instruments with myocarditis and COVID-19 infection. Association of the genetic instruments with myocarditis and severe COVID-19. Association of the genetic instruments with pericarditis and hospitalized COVID-19. Association of the genetic instruments with pericarditis and COVID-19 infection. Association of the genetic instruments with pericarditis and severe COVID-19. [file Datasheet2.zip › Data Sheet 2_v2/Data Sheet 2 (1)/Supplementary figures (1-12)/Figure 5.tif]

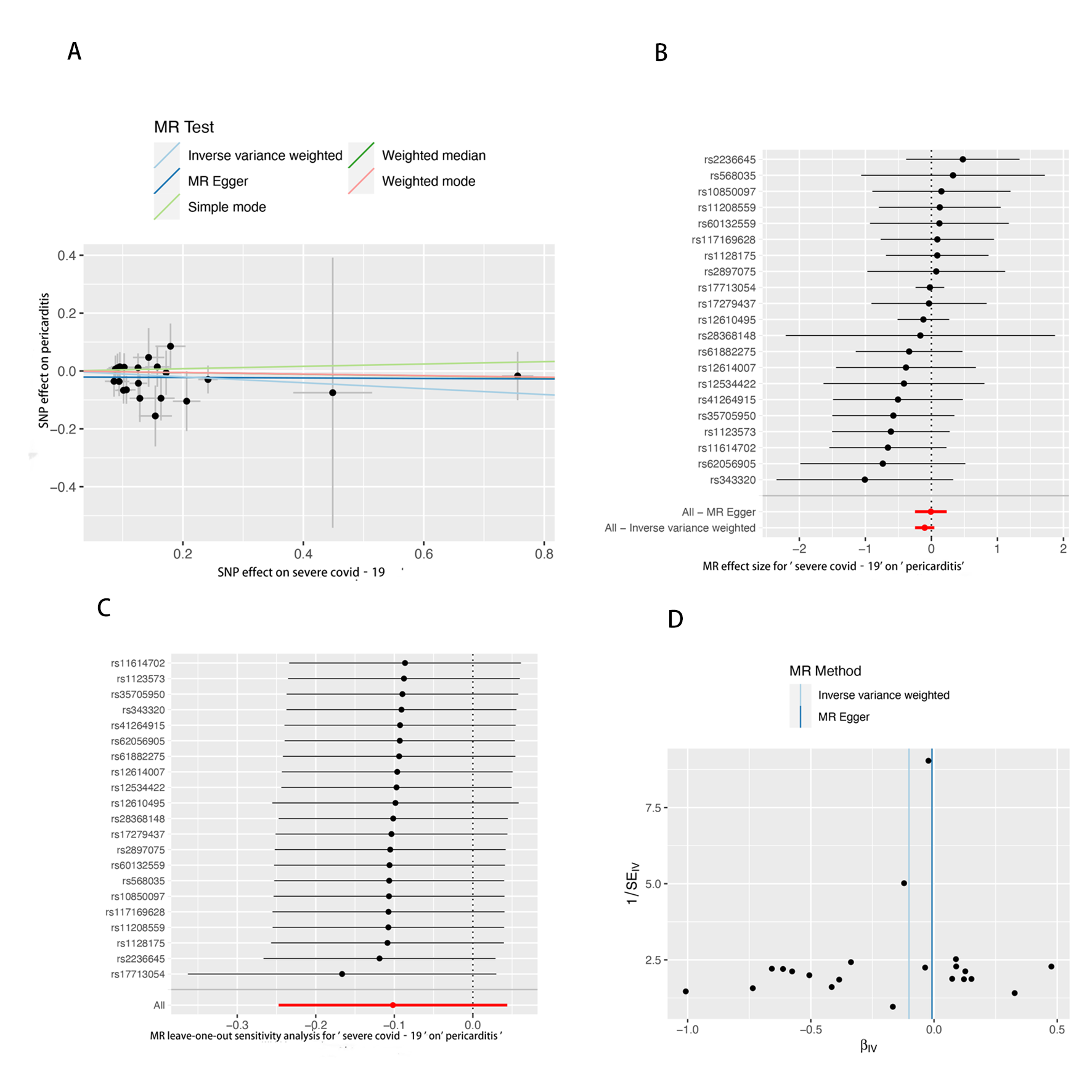

Supplement: Supplementary Table S1, Supplementary Table S2, Supplementary Table S3, Supplementary Table S4, Supplementary Table S5, Supplementary Table S6, Supplementary Table S7, Supplementary Table S8, Supplementary Table S9, Supplementary Table S10, Supplementary Table S11, Supplementary Table S12 — Association of the genetic instruments with severe COVID-19 and myocarditis. Association of the genetic instruments with severe COVID-19 and pericarditis. Association of the genetic instruments with hospitalized COVID-19 and myocarditis. Association of the genetic instruments with hospitalized COVID-19 and pericarditis. Association of the genetic instruments with COVID-19 infection and myocarditis. Association of the genetic instruments with COVID-19 infection and pericarditis. Association of the genetic instruments with myocarditis and hospitalized COVID-19. Association of the genetic instruments with myocarditis and COVID-19 infection. Association of the genetic instruments with myocarditis and severe COVID-19. Association of the genetic instruments with pericarditis and hospitalized COVID-19. Association of the genetic instruments with pericarditis and COVID-19 infection. Association of the genetic instruments with pericarditis and severe COVID-19. [file Datasheet2.zip › Data Sheet 2_v2/Data Sheet 2 (1)/Supplementary figures (1-12)/Figure 6.tif]

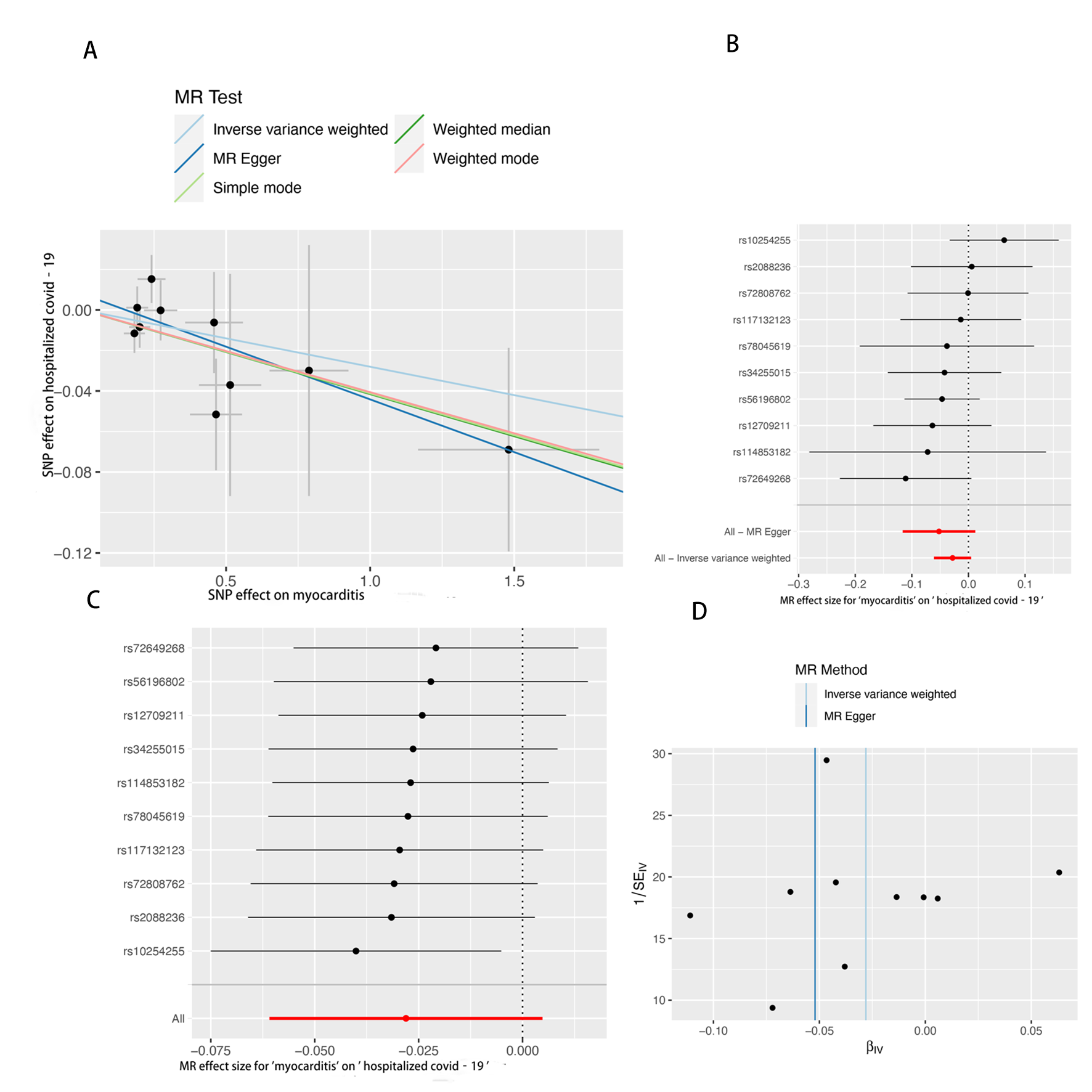

Supplement: Supplementary Table S1, Supplementary Table S2, Supplementary Table S3, Supplementary Table S4, Supplementary Table S5, Supplementary Table S6, Supplementary Table S7, Supplementary Table S8, Supplementary Table S9, Supplementary Table S10, Supplementary Table S11, Supplementary Table S12 — Association of the genetic instruments with severe COVID-19 and myocarditis. Association of the genetic instruments with severe COVID-19 and pericarditis. Association of the genetic instruments with hospitalized COVID-19 and myocarditis. Association of the genetic instruments with hospitalized COVID-19 and pericarditis. Association of the genetic instruments with COVID-19 infection and myocarditis. Association of the genetic instruments with COVID-19 infection and pericarditis. Association of the genetic instruments with myocarditis and hospitalized COVID-19. Association of the genetic instruments with myocarditis and COVID-19 infection. Association of the genetic instruments with myocarditis and severe COVID-19. Association of the genetic instruments with pericarditis and hospitalized COVID-19. Association of the genetic instruments with pericarditis and COVID-19 infection. Association of the genetic instruments with pericarditis and severe COVID-19. [file Datasheet2.zip › Data Sheet 2_v2/Data Sheet 2 (1)/Supplementary figures (1-12)/Figure 7.tif]

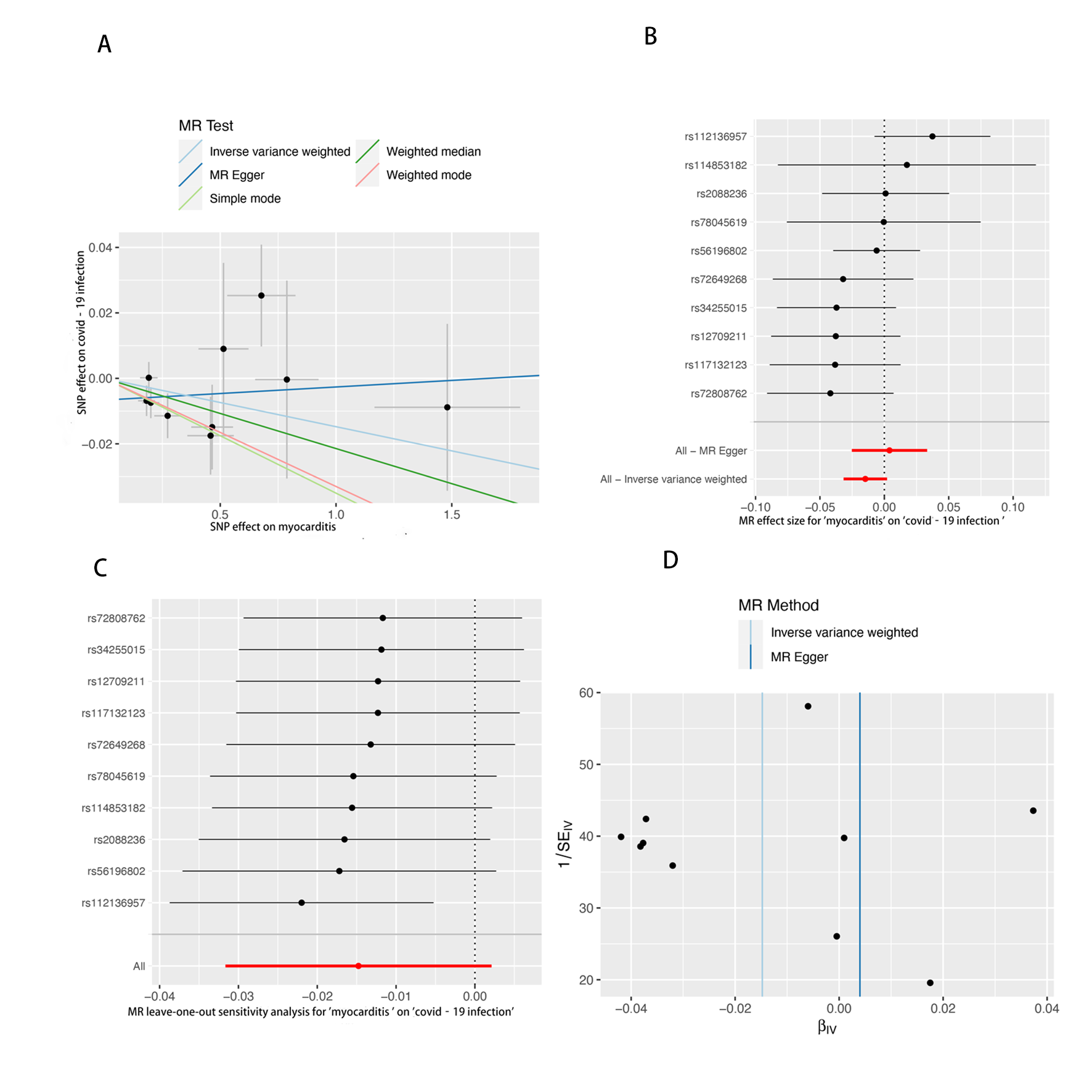

Supplement: Supplementary Table S1, Supplementary Table S2, Supplementary Table S3, Supplementary Table S4, Supplementary Table S5, Supplementary Table S6, Supplementary Table S7, Supplementary Table S8, Supplementary Table S9, Supplementary Table S10, Supplementary Table S11, Supplementary Table S12 — Association of the genetic instruments with severe COVID-19 and myocarditis. Association of the genetic instruments with severe COVID-19 and pericarditis. Association of the genetic instruments with hospitalized COVID-19 and myocarditis. Association of the genetic instruments with hospitalized COVID-19 and pericarditis. Association of the genetic instruments with COVID-19 infection and myocarditis. Association of the genetic instruments with COVID-19 infection and pericarditis. Association of the genetic instruments with myocarditis and hospitalized COVID-19. Association of the genetic instruments with myocarditis and COVID-19 infection. Association of the genetic instruments with myocarditis and severe COVID-19. Association of the genetic instruments with pericarditis and hospitalized COVID-19. Association of the genetic instruments with pericarditis and COVID-19 infection. Association of the genetic instruments with pericarditis and severe COVID-19. [file Datasheet2.zip › Data Sheet 2_v2/Data Sheet 2 (1)/Supplementary figures (1-12)/Figure 8.tif]

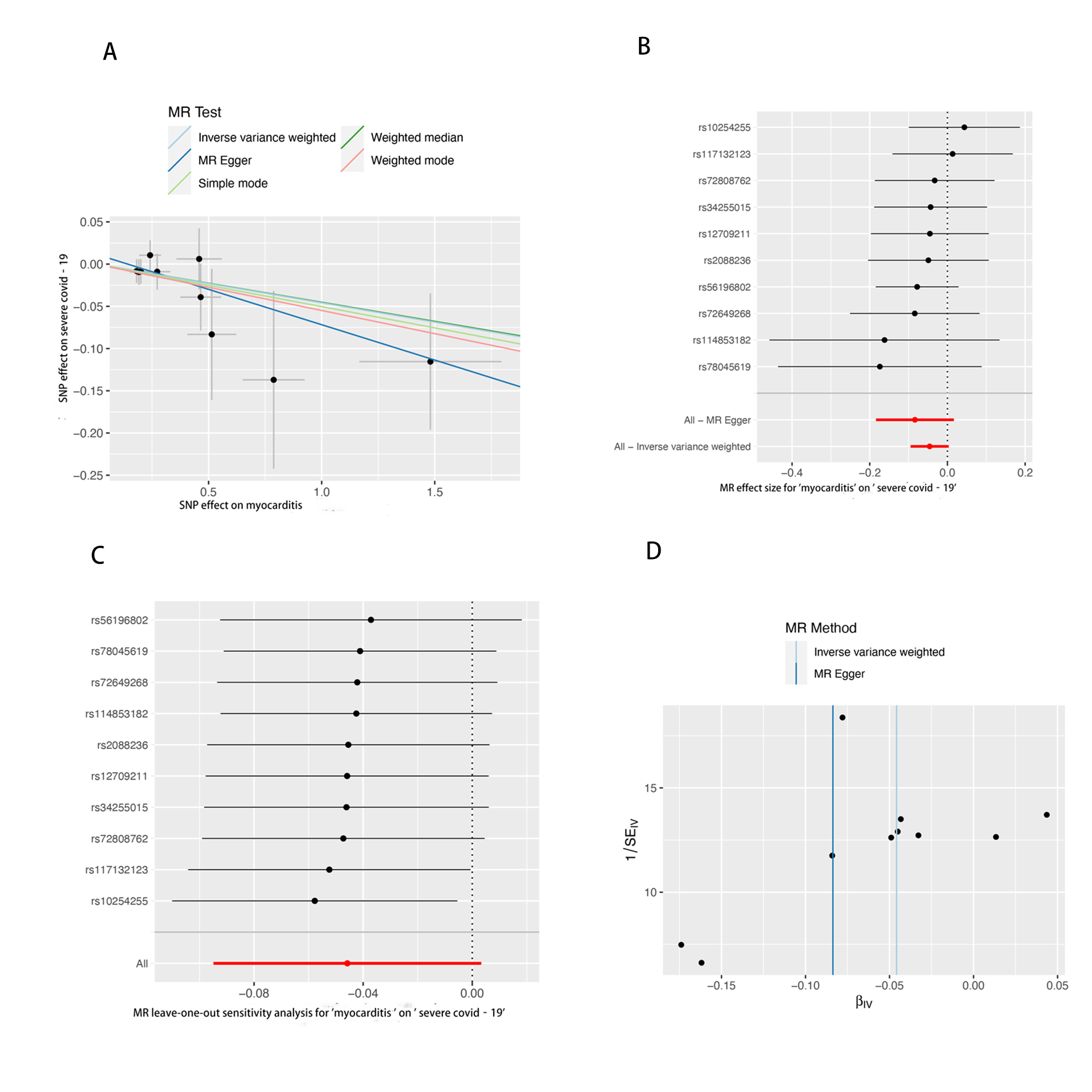

Supplement: Supplementary Table S1, Supplementary Table S2, Supplementary Table S3, Supplementary Table S4, Supplementary Table S5, Supplementary Table S6, Supplementary Table S7, Supplementary Table S8, Supplementary Table S9, Supplementary Table S10, Supplementary Table S11, Supplementary Table S12 — Association of the genetic instruments with severe COVID-19 and myocarditis. Association of the genetic instruments with severe COVID-19 and pericarditis. Association of the genetic instruments with hospitalized COVID-19 and myocarditis. Association of the genetic instruments with hospitalized COVID-19 and pericarditis. Association of the genetic instruments with COVID-19 infection and myocarditis. Association of the genetic instruments with COVID-19 infection and pericarditis. Association of the genetic instruments with myocarditis and hospitalized COVID-19. Association of the genetic instruments with myocarditis and COVID-19 infection. Association of the genetic instruments with myocarditis and severe COVID-19. Association of the genetic instruments with pericarditis and hospitalized COVID-19. Association of the genetic instruments with pericarditis and COVID-19 infection. Association of the genetic instruments with pericarditis and severe COVID-19. [file Datasheet2.zip › Data Sheet 2_v2/Data Sheet 2 (1)/Supplementary figures (1-12)/Figure 9.tif]
